# Low-grade inflammation, diet composition and health: current research evidence and its translation

**DOI:** 10.1017/S0007114515002093

**Published:** 2015-07-31

**Authors:** Anne M. Minihane, Sophie Vinoy, Wendy R. Russell, Athanasia Baka, Helen M. Roche, Kieran M. Tuohy, Jessica L. Teeling, Ellen E. Blaak, Michael Fenech, David Vauzour, Harry J. McArdle, Bas H. A. Kremer, Luc Sterkman, Katerina Vafeiadou, Massimo Massi Benedetti, Christine M. Williams, Philip C. Calder

**Affiliations:** 1 Department of Nutrition, Norwich Medical School, University of East Anglia, NorwichNR4 7TJ, UK; 2 Mondelēz International – R&D, Nutrition Department, 91400Saclay, France; 3 Rowett Institute of Nutrition and Health, University of Aberdeen, AberdeenAB21 9SB, UK; 4 Formerly ILSI Europe a.i.s.b.l., Avenue E. Mounier 83, Box 6, B-1200Brussels, Belgium; 5 Nutrigenomics Research Group, UCD Institute of Food and Health and UCD Conway Institute, Belfield, University College Dublin, Dublin 4, Republic of Ireland; 6 Department of Food Quality and Nutrition, Research and Innovation Centre, Fondazione Edmund Mach, San Michele all'Adige, 38010Trento, Italy; 7 Centre for Biological Sciences, Faculty of Natural and Environmental Sciences, University of Southampton, SouthamptonSO16 6YD, UK; 8 Department of Human Biology, NUTRIM School for Nutrition, Toxicology and Metabolism, Maastricht University, Maastricht, The Netherlands; 9 Nutrigenomics and Neurodegenerative Disease Prevention, Preventative Health Flagship, CSIRO, Animal, Food and Health Sciences, Adelaide, Australia; 10 Microbiology and Systems Biology, TNO, Zeist, 3704HE, The Netherlands; 11 Newtricious R&D B.V., Oirlo, 5808AL, The Netherlands; 12 School of Life and Medical Sciences, University of Hertfordshire, HatfieldAL10 9AB, UK; 13 Department of Internal Medicine, University of Perugia, Perugia, Italy; 14 Hugh Sinclair Unit of Human Nutrition, Department of Food and Nutritional Sciences, University of Reading, ReadingRG6 6AP, UK; Faculty of Medicine, University of Southampton, SouthamptonSO16 6YD, UK; 16 NIHR Southampton Biomedical Research Centre, Southampton University Hospital NHS Foundation Trust and University of Southampton, SouthamptonSO16 6YD, UK

**Keywords:** Low-grade inflammation, Biomarkers, Chronic diseases, Health claims

## Abstract

The importance of chronic low-grade inflammation in the pathology of numerous age-related chronic conditions is now clear. An unresolved inflammatory response is likely to be involved from the early stages of disease development. The present position paper is the most recent in a series produced by the International Life Sciences Institute's European Branch (ILSI Europe). It is co-authored by the speakers from a 2013 workshop led by the Obesity and Diabetes Task Force entitled ‘Low-grade inflammation, a high-grade challenge: biomarkers and modulation by dietary strategies’. The latest research in the areas of acute and chronic inflammation and cardiometabolic, gut and cognitive health is presented along with the cellular and molecular mechanisms underlying inflammation–health/disease associations. The evidence relating diet composition and early-life nutrition to inflammatory status is reviewed. Human epidemiological and intervention data are thus far heavily reliant on the measurement of inflammatory markers in the circulation, and in particular cytokines in the fasting state, which are recognised as an insensitive and highly variable index of tissue inflammation. Potential novel kinetic and integrated approaches to capture inflammatory status in humans are discussed. Such approaches are likely to provide a more discriminating means of quantifying inflammation–health/disease associations, and the ability of diet to positively modulate inflammation and provide the much needed evidence to develop research portfolios that will inform new product development and associated health claims.

## Introduction and overview of the focus of the position paper

Inflammation is a central component of innate (non-specific) immunity. In generic terms, inflammation is a local response to cellular injury that is marked by increased blood flow, capillary dilatation, leucocyte infiltration, and the localised production of a host of chemical mediators, which serves to initiate the elimination of toxic agents and the repair of damaged tissue^(^ ^1^ ^)^. It is now clear that the termination (alternatively known as resolution) of inflammation is an active process involving cytokines and other anti-inflammatory mediators, particularly lipids, rather than simply being the switching off of pro-inflammatory pathways^(^ ^2^ ^,^ ^3^ ^)^.

Inflammation acts as both a ‘friend and foe’: it is an essential component of immunosurveillance and host defence, yet a chronic low-grade inflammatory state is a pathological feature of a wide range of chronic conditions, such as the metabolic syndrome (MetS), non-alcoholic fatty liver disease (NAFLD), type 2 diabetes mellitus (T2DM) and CVD^(^ ^4^ ^,^ ^5^ ^)^. Although the association between inflammation and chronic conditions is widely recognised, the issue of causality and the degree to which inflammation contributes and serves as a risk factor for the development of disease remain unresolved. As will be discussed, part of this uncertainty is due to a general lack of sensitive and specific biomarkers of low-grade chronic inflammation that can be used in human trials^(^ ^1^ ^)^.

The present article results from an International Life Sciences Institute (ILSI) Europe Workshop held in September 2013 in Granada, Spain entitled ‘Low-grade inflammation a high grade challenge: biomarkers and modulation by dietary strategies’, and aims to serve as an update to existing reviews in the area of inflammation and health and its assessment and modulation^(^ ^1^ ^,^ ^6^ ^,^ ^7^ ^)^. In particular, the present article will focus on the latest research findings in the areas of inflammation and cardiometabolic, cognitive and gut health, and how early-life nutrition and the macronutrient and plant bioactive composition of the adult diet influence inflammatory processes. It will discuss existing and emerging methods used to quantify inflammatory status in humans. Importantly, the article will identify knowledge gaps and methodological limitations that need to be addressed.

## Exploring the role of inflammation in health and chronic diseases

### Low-grade inflammation in cardiometabolic disease

The role of inflammation in the early-stage pathophysiology of atherothrombotic events has been recognised for over 20 years. Leucocyte recruitment into the sub-endothelial compartment of damaged arteries initiates a cascade of events mediated by leucocyte-derived inflammatory mediators. In particular, chemokines and cytokines propagate atherosclerosis via (1) increased chemokine production and expression of endothelial adhesion molecules, stimulating further leucocyte recruitment, (2) promoting lipid-laden foam-cell formation, (3) initiating smooth muscle cell proliferation, and (4) inducing plaque instability and eventual rupture^(^ ^8^ ^,^ ^9^ ^)^. The ensuing thrombosis is also in large part dependent on the inflammatory status of the ruptured plaque.

In addition to a direct role on events within the arterial wall, inflammation is an important determinant of the multi-organ cardiometabolic dysfunction, and the increased risk of T2DM, NAFLD and CVD associated with obesity^(^ ^10^ ^)^. Adipose tissue hypertrophy is associated with immune cell infiltration, in particular that of macrophages and T cells, and a local pro-inflammatory milieu wherein key cytokines including TNF-α, IL-6 and IL-1β impede the insulin signalling cascade to induce insulin resistance^(^ ^11^ ^,^ ^12^ ^)^. This ultimately leads to a dysregulation of glucose and lipid metabolism in adipose tissue, skeletal muscle and liver. However, up to 30 % of obese individuals are considered metabolically healthy (MHO)^(^ ^13^ ^)^, and there is evidence to suggest that a lack of the typical elevation in the inflammatory profile associated with obesity may underlie this ‘protected’ MHO phenotype. For example, in morbidly obese individuals, Barbarroja and co-workers observed mean homeostatic model assessment for insulin resistance (HOMA-IR) scores (insulin sensitivity index) of 3·31 and 11·48 in subjects with MHO (BMI 55 kg/m^2^) or who were metabolically unhealthy obese (BMI 56 kg/m^2^), respectively, which was associated with a 2- to 4-fold greater adipose expression of inflammatory cytokines (TNF-α, IL-1β and IL-6) between the two obese groups^(^ ^14^ ^)^.

**Fig. 1:**
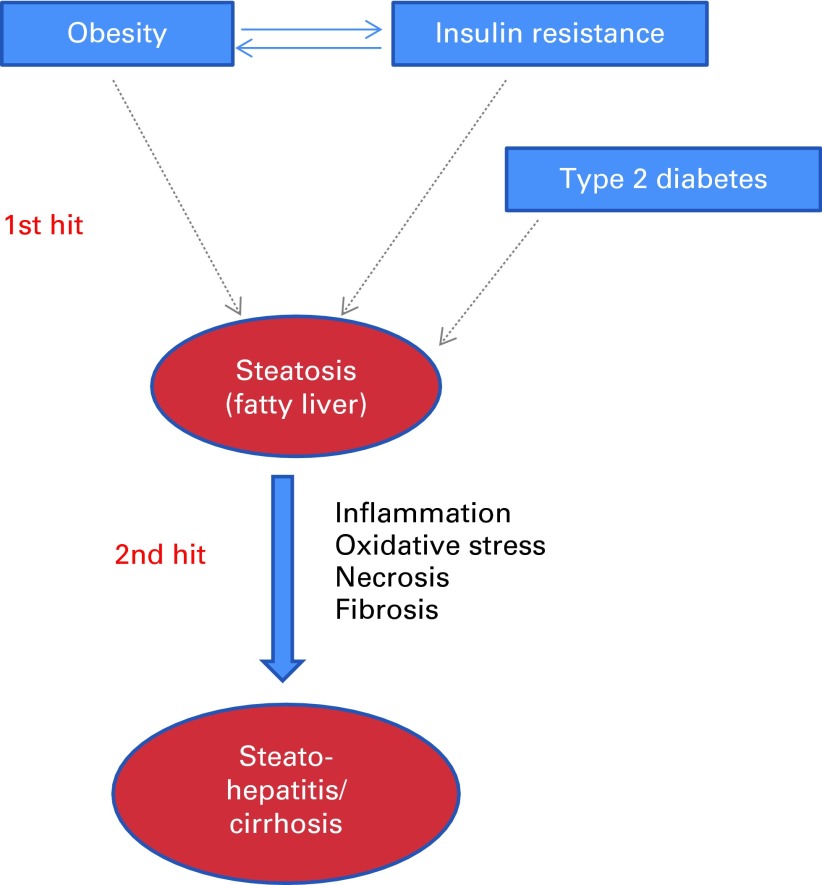
Two-hit model of non-alcoholic fatty liver disease. (A colour version of this figure can be found online at http://www.journals.cambridge.org/bjn).

Inflammation plays a direct role in the progression of NAFLD, the most common liver disorder in Western countries. NAFLD comprises a spectrum of conditions ranging from benign steatosis to non-alcoholic steatohepatitis characterised by hepatocyte injury (hepatocyte ballooning and Mallory bodies) and necroinflammation, and potentially to progressive fibrosis that can lead to cirrhosis^(^ ^15^ ^,^ ^16^ ^)^. The pathological progression of NAFLD is considered to have a two-hit basis (Fig. 1). The first hit, hepatocyte accumulation of fat, is thought to arise due to an increased delivery of fatty acids to the hepatocyte, an increase in hepatocyte fatty acid and TAG synthesis, and decreased fatty acid oxidation. The resultant excess of fat may result in lipotoxicity and a pro-inflammatory and pro-oxidative state (the second hit), which ultimately induces cellular senescence, which, if unchecked, leads to fibrosis and cirrhosis. Hepatic inflammation is mediated via the activation of local macrophages called Kupffer cells. Currently, no medication or surgical procedure has been approved for treating NAFLD or non-alcoholic steatohepatitis with confidence. Considering the overall lack of success in curbing global trends in the prevalence of excess body weight, inflammatory processes are emerging as a strong therapeutic target to reduce the risk of T2DM, CVD and NAFLD in obese individuals.

### Gut–systemic inflammatory associations

With recent significant advances in the ability to characterise the gut microbiota in increasing detail, comes the recognition of the importance of the microbiota not only in gastrointestinal health, but also in systemic metabolism and cardiometabolic health, with the immune system and inflammatory processes central to gut–systemic tissue ‘cross-talk’. The human intestine contains 1 × 10^13^ to 1 × 10^14^ bacterial cells, which outnumber human cells by a factor of 10 to 1 and contain approximately 150 times as many genes as the human genome^(^ ^17^ ^)^. Increasing evidence indicates that the microbiota is significantly altered through the ageing process^(^ ^18^ ^,^ ^19^ ^)^ and in obesity^(^ ^18^ ^)^, with a deleterious decline in microbiota ‘richness’ and gene expression diversity evident in both situations^(^ ^18^ ^)^.

Gastrointestinal tract–microbiota interactions influence host health, and in particular immune function, by promoting the development and maintenance of the mucosal immune system, protecting against pathogen invasion and maintaining gastrointestinal tract barrier integrity^(^ ^20^ ^)^. Gut permeability to bacterial lipopolysaccharides (LPS), a potent inflammatory stimulant, appears to be an important trigger for low-grade systemic inflammation. LPS are found on the outer membrane of Gram-negative bacteria such as Proteobacteria (e.g. *Escherichia coli*), and serve as an endotoxin. In the elderly, a higher count of LPS-producing bacteria in the colon, along with a lower abundance of bifidobacteria^(^ ^21^ ^,^ ^22^ ^)^, a combination which is thought to promote increased gut permeability^(^ ^21^ ^)^, is likely to lead to higher plasma levels of LPS (termed metabolic endotoxaemia). Through the interaction with Toll-like receptor 4 on mononuclear cells, microbiota-derived LPS may be an important trigger in the development of inflammation and metabolic diseases^(^ ^23^ ^)^. In a recent dietary intervention study in male C57Bl/6 mice, the alteration in microbiota profiles as a result of a high-fat diet was strongly associated with gut permeability, endotoxaemia and adipose tissue inflammation^(^ ^24^ ^)^.

In addition to its role in low-grade inflammatory cardiometabolic conditions, emerging evidence is suggesting that the gut microbiota can influence the risk of high-grade autoimmune inflammatory conditions such as type 1 diabetes mellitus, coeliac disease, inflammatory bowel disease and rheumatoid arthritis^(^ ^25^ ^–^ ^27^ ^)^, the incidence of which has risen dramatically since the 1940s. These conditions are now thought to affect 5–10 % of those in Western societies^(^ ^28^ ^)^. Certain members of the gut microbiota have been shown to induce mimics of human antigens and trigger the production of autoantibodies responsible for aberrant immune responses to normal human proteins and hormones including leptin, peptide YY and ghrelin^(^ ^29^ ^)^. It is not unreasonable to speculate that the adverse impact of the energy-dense, nutrient-poor Western-style diet on human gut microbiota and immune system, which have both been finely tuned and honed by high-fibre, high-polyphenol traditional diets over the millennia, may therefore be an important contributor to the environmental stimuli that trigger and progress autoimmune conditions^(^ ^30^ ^)^. A possible starting point when discussing the underlying mechanisms by which diets rich in whole plant foods or fermentable fibres can have an impact on immune function and tolerance may be the recent demonstration that butyrate, an important fermentation end product produced by the gut microbiota from fibre, controls human dendritic cell maturation, a key process in immune homeostasis, since dendritic cells are considered as ‘gate keepers’ of the immune system^(^ ^31^ ^,^ ^32^ ^)^. In addition, butyrate induces murine peripheral regulatory T-cell generation^(^ ^33^ ^)^, acetate affects neutrophil chemotaxis and oxidative burst, butyrate inhibits adipocyte–macrophage inflammatory interactions^(^ ^34^ ^)^, and propionate reduces the inflammatory output of adipose tissue^(^ ^35^ ^)^. Probiotic, fibre or polyphenol up-regulation of microbial activities that control both the quantity and profile of bile acids returning to the liver via the enterohepatic circulation with their subsequent regulation of farnesoid X receptor and TGR5 is also emerging as an important pathway linking the gut microbiota with extra-intestinal physiological/immune function^(^ ^33^ ^,^ ^36^ ^,^ ^37^ ^)^.

### Low-grade systemic inflammation and neuroinflammation

Communication between the systemic immune system and the central nervous system (CNS) is a critical but often overlooked component of the inflammatory response to tissue injury, disease or infection. Activation of highly conserved neuronal and hormonal communication pathways in mammals drives diverse CNS-regulated components of the inflammatory response, including fever, neurogenic inflammation, descending anti-inflammatory mechanisms and a coordinated set of metabolic and behavioural changes, including fatigue, anhedonia, depression and mild cognitive impairment. These behavioural changes are collectively referred to as ‘sickness behaviour’^(^ ^38^ ^–^ ^40^ ^)^. Experimental studies have provided evidence that activation of microglia, the macrophages of the CNS, as well as the cerebral vasculature, plays a key role in the development of these behavioural changes, by inducing pro-inflammatory mediators, such as IL-1β, TNF-α and PGE_2_ in the CNS^(^ ^38^ ^,^ ^41^ ^,^ ^42^ ^)^.

Much of what we know is derived from studies using mimetics of bacterial and viral infection. Depending on the stimulus used, these mimetics induce a transient response in otherwise healthy subjects; for example, administration of LPS results in enhanced production of IL-6 (approximately 80-fold) and IL-1β (approximately 4-fold), peaking at 3 h after a challenge and returning to baseline at 24 h^(^ ^32^ ^)^. CNS responses to (patho)physiological stimuli, such as genuine infections or low-grade inflammation as a result of the MetS, are less well described.

Development of sickness behaviour in response to an infection is part of the normal response to fighting infection, and can occur during low-grade sub-pyrogenic inflammation^(^ ^41^ ^)^; however, these adaptive responses are not always harmless. Microglia have a very low turnover, and it has been suggested that these long-lived cells have an innate memory, resulting in a prolonged and heightened response under neuroinflammatory conditions^(^ ^43^ ^)^. A normal part of the homeostatic signalling from the periphery to the brain, therefore, has the potential to have a profound impact on brain disease initiation or progression^(^ ^44^ ^,^ ^45^ ^)^. In a recent prospective clinical study, Alzheimer's disease patients were followed for 6 months and assessed for the presence of circulating cytokines, episodes of microbial infection and cognitive decline. Patients with both high levels of TNF-α (>4·2 pg/ml) at baseline and microbial infection during the assessment period showed a 4-fold greater cognitive decline, relative to patients with low levels of TNF-α ( < 2·4 ng/ml) at baseline and no infections^(^ ^46^ ^)^. Raised serum levels of TNF-α and IL-6, but not CRP, are also associated with increased frequency of other common neuropsychiatric symptoms observed in Alzheimer's disease patients, including apathy, anxiety, depression and agitation^(^ ^47^ ^)^.

Recently, the effects of LPS and a real infection (*Salmonella typhimurium*) on cerebral endothelial and microglial activation were compared. While LPS administration resulted in a robust but transient neuroinflammatory response, a genuine infection induced a prolonged pro-inflammatory cytokine response in the CNS, leading to microglial priming^(^ ^48^ ^)^.

A detailed consideration of the impact and mechanistic basis for the association between neuroinflammation and neuronal and overall CNS function, cognition and the risk of age-related cognitive decline and dementia is outside the scope of the present review, and has been the topic of many recent expert review articles^(^ ^49^ ^–^ ^54^ ^)^.

Collectively, these data highlight inflammatory pathways as important targets for strategies promoting healthy brain ageing and reducing the risk of age-related cognitive decline.

## Dietary modulation of low-grade inflammation

There is a substantial amount of evidence to suggest that many foods, nutrients and non-nutrient food components modulate inflammation both acutely and chronically^(^ ^1^ ^,^ ^6^ ^)^. However, dietary studies have been typically limited to measuring a small number of blood markers of inflammation, often in the fasting state, and these may not necessarily reflect inflammation in tissue compartments or what happens in response to inflammatory challenges. This presents a significant limitation to our understanding of diet/nutrient–inflammation interactions. Previous ILSI Europe activities have dealt extensively with the food/nutrition–inflammation interaction^(^ ^6^ ^,^ ^7^ ^)^, and it is beyond the scope of the present review to provide a systematic or extensive coverage of this area. Instead, some specific examples will be discussed.

### Dietary fats and inflammation

Dietary fatty acids may affect inflammatory processes through effects on body weight and adipose tissue mass and via an impact on membrane and lipid raft composition and function. Within the cell, membrane-derived fatty acids and their derivatives can influence inflammation by serving as modulators of NF-κB and PPAR-α/γ transcription factor pathways^(^ ^55^ ^)^, and as precursors for a host of eicosanoid and docosanoid oxidation products produced via the action of epoxygenases, lipoxygenases and cyclo-oxygenases^(^ ^56^ ^)^. Also, recent advances in the field have uncovered NLRP3 (NACHT, LRR and PYD domains-containing protein 3) inflammasome activation and IL-1β signalling as a key sensor of SFA-mediated metabolic stress in obesity and T2DM^(^ ^57^ ^)^ and EPA- and DHA-derived resolvins and protectins that actively ameliorate a pro-inflammatory state^(^ ^58^ ^)^. Obesity significantly reduced DHA-derived 17-hydroxydocosahexaenoic acid, a resolvin D1 precursor, and protectin D1 in adipose tissue, which may in turn have pro-inflammatory consequences^(^ ^59^ ^)^. Also, dietary EPA/DHA supplementation within an obesogenic dietary challenge restored endogenous adipose resolvin and protectin biosynthesis, concomitant with attenuated adipose inflammation and insulin resistance^(^ ^59^ ^)^. An elegant human study showed that a relatively high dose of LC *n*-3 PUFA augmented anti-inflammatory eicosanoid secretion and attenuated inflammatory gene expression in the subcutaneous adipose tissue of severely obese non-diabetic patients^(^ ^60^ ^)^. Thus, there is much recent information on novel mechanisms of action by which dietary fatty acids of different classes influence inflammatory processes, some acting in pro-inflammatory and others in anti-inflammatory or inflammation-resolving ways.

There is some evidence, albeit not always consistent, for pro-inflammatory effects of dietary SFA^(^ ^1^ ^)^. Much of this evidence comes from either *in vitro* or cross-sectional studies, and there are limited randomised controlled trial (RCT) examining changes in SFA intake and inflammation in humans. The LIPGENE RCT investigated the effects of substituting dietary SFA with MUFA or as part of a low-fat diet, with or without LC *n*-3 PUFA supplementation, in subjects with the MetS^(^ ^61^ ^)^. While a low-fat *n*-3 PUFA-enriched diet significantly reduced the risk of the MetS^(^ ^62^ ^)^, modifying dietary fat had no significant effect on key biomarkers of cardiometabolic risk including insulin sensitivity and the plasma inflammatory markers assessed^(^ ^63^ ^)^. However, there was clear modulation of NF-κB-mediated inflammation and oxidative stress in the postprandial state according to lipid composition^(^ ^64^ ^,^ ^65^ ^)^. This lack of impact of LC *n*-3 PUFA on the fasting plasma inflammasome in humans^(^ ^66^ ^)^ is in line with previous human studies^(^ ^63^ ^,^ ^67^ ^)^, but contradicts the effects observed in a wide variety of cell and animal models. However, as will be discussed in the section ‘Translating research into public health benefit and novel products’, it is difficult to know whether the output from these RCT truly demonstrates a lack of efficacy or reflects insufficient dose and/or duration or poor selection of fasting plasma biomarkers of inflammation, which are insensitive to physiologically meaningful changes occurring in key metabolic tissues such as the liver and adipose tissue.

As with other common phenotypes, there is evidence emerging that the associations between dietary fat composition and inflammation are influenced by common gene variants^(^ ^68^ ^)^. In the LIPGENE study, SNP in the genes encoding the anti-inflammatory peptide adiponectin (ADIPOQ) and its receptor (ADIPOR1) have been shown to interact with SFA to modulate the effect of dietary fat modification on insulin resistance^(^ ^69^ ^)^, and using a case–control approach, it was observed that a common SNP of the *C3* gene was related to the risk of the MetS, but more importantly, the impact of this was greatly accentuated by high plasma levels of SFA^(^ ^70^ ^)^. Also, the combination of polymorphisms in genes encoding IL-6, lymphotoxin α (LTA) and TNF-α had an additive effect, which interacted with plasma fatty acid status to modulate the risk of the MetS^(^ ^71^ ^)^. Grimble *et al.* ^(^ ^72^ ^)^ demonstrated that the ability of LC *n*-3 PUFA to decrease TNF-α production is influenced by inherent TNF-α production and by polymorphisms in the *TNF-α* and *LTA* genes.

Inflammation in the postprandial state is likely to contribute to the pathological impact of exaggerated postprandial lipaemia^(^ ^73^ ^)^. Although there has been some investigation of the impact of meal fatty acid composition on non-fasting inflammatory biomarkers, the data thus far remain inconsistent^(^ ^73^ ^)^. It has been reported that in overweight men, plasma IL-6, TNF-α and soluble vascular adhesion molecule-1 concentrations decreased after an *n*-6 PUFA-rich meal, while markers were increased after a SFA-rich meal^(^ ^74^ ^)^. In contrast, Manning *et al.* ^(^ ^75^ ^)^ showed that high-fat meals increased IL-6, independent of the type of fatty acid, and had no impact on IL-8 and TNF-α concentrations.

### Dietary carbohydrates and inflammation

Besides postprandial lipaemia, postprandial glucose is an independent predictor of diabetes and CVD, an effect which may be mediated through oxidative stress and inflammation^(^ ^76^ ^)^. Importantly, there appears to be no glycaemic threshold for reduction of either microvascular or macrovascular complications. The progressive relationship between plasma glucose and the risk of CVD extends well below the diabetic threshold^(^ ^77^ ^,^ ^78^ ^)^.

Acute glucose variations from peaks to nadirs include postprandial glucose excursions that can be described by two components. The first component, which is the duration of the postprandial glucose increment, is a major contributor to chronic sustained hyperglycaemia, while the second component, which is the magnitude of the postprandial rise, is more often a reflection of glucose variability. It is difficult to discriminate between the contributions of these two components of dysglycaemia. It seems that both contribute to the two main mechanisms that lead to diabetic and cardiovascular complications, namely excessive protein glycation and activation of oxidative stress and inflammation.

Although mechanistic evidence indicates a positive correlation between the glycaemic index and load of the diet and low-grade inflammation, intervention studies, to date, do not convincingly support this. Hu *et al.* ^(^ ^79^ ^)^ observed a stepwise relationship between dietary glycaemic index and oxidative stress markers in healthy adults. Furthermore, high-glycaemic index carbohydrates increase NF-κB activation and NF-κB binding in mononuclear cells of young, lean healthy subjects^(^ ^80^ ^)^. Diets low in glycaemic load and high in whole grains may have a protective effect against systemic inflammation in diabetic patients, as reviewed elsewhere^(^ ^81^ ^)^. Consistent with this, epidemiological studies have shown an inverse relationship between dietary fibre and CRP levels. Both the DASH diet (naturally high in fibre, i.e. 30 g fibre/d) and a fibre-supplemented usual diet (30 g psyllium fibre/d) decreased CRP concentrations in lean normotensive subjects^(^ ^82^ ^)^. In contrast, a high-carbohydrate, low-fat diet with a relatively high dietary fibre and complex carbohydrate content, within the context of a lifestyle intervention programme, has been shown to reduce diabetes incidence in the long term by 50 %^(^ ^83^ ^)^. The prominent role of the type of carbohydrate has also been illustrated in studies showing that dietary carbohydrate modification, i.e. an oat/wheat/potato diet, up-regulated sixty-two genes related to stress, cytokine–chemokine-mediated immunity and IL pathways compared with a rye–pasta diet^(^ ^84^ ^)^. These differences in the inflammatory response have been ascribed to differences in the early insulin response and the resultant late hypoglycaemia in the oat/wheat/potato group.

Taken together, studies have suggested that healthy eating patterns characterised by reduced postprandial glycaemia and lipaemia are associated with reduced concentrations of markers of low-grade inflammation.

### Plant bioactive compounds and inflammation

Recent prospective cohort data suggest that improved cognitive function and a reduced risk of age-related neurodegenerative diseases, associated with increased fruit and vegetable intake^(^ ^85^ ^–^ ^87^ ^)^, may be in large part attributable to intake of specific flavonoids^(^ ^87^ ^)^, and may involve an effect on inflammatory processes (Table 1). In particular, increased consumption of total flavonoids was positively associated with episodic memory in middle-aged adults^(^ ^88^ ^)^ and with a reduced rate of cognitive decline in adults aged 70 years and over^(^ ^89^ ^)^. The anthocyanin group of flavonoids, with certain soft fruits providing the most significant dietary source, has emerged as being particularly potent. In the Nurses' Health Cohort, greater intakes of blueberries and strawberries were associated with slower rates of cognitive decline, with a high intake of soft fruits estimated to delay cognitive ageing by up to 2·5 years^(^ ^90^ ^)^. Furthermore, a large cross-sectional study has also indicated that total flavonoid intake is inversely correlated with serum CRP concentrations^(^ ^91^ ^)^. In support of this association, a number of dietary intervention studies have provided evidence that dietary flavonoids are capable of modulating inflammatory cytokines (e.g. TNF-α) and CRP production^(^ ^91^ ^–^ ^94^ ^)^. However, there are relatively few human RCT investigating the anti-inflammatory and cognitive effects of flavonoids (Table 1).

**Table 1:** Dietary flavonoids and inflammation: evidence from epidemiological and intervention studies

Reference	Subject description	Product	Dosage/duration of intake	Results
Chun *et al.* ^(^ ^91^ ^)^	8335 men and women, age 19 years and over	Total flavonoid	Dietary intake	Reduced plasma concentrations of CRP (25–30 %) in flavonoid consumers *v*. non-consumers
Landberg *et al.* ^(^ ^139^ ^)^	1194–1598 women	Flavonoid-rich foods	Dietary intake	Reduced plasma concentrations of CRP (21 %), IL-18 (20 %) and TNF-R2 (8 %) for ≥ 1 servings/d *v*. < 1 serving/month
Edirisinghe *et al.* ^(^ ^140^ ^)^	Twenty-four overweight men and women	Strawberry anthocyanins	Single dose/6 h	Reduced concentrations of CRP (13 %) and IL-6 (16 %), 6 h following a high-carbohydrate, moderate-fat meal, but no results observed for TNF-α and IL-1β
Steptoe *et al.* ^(^ ^141^ ^)^	Thirty-seven non-smoking men, age 18–55 years	Black tea	1050 mg tea extract/6 weeks	Decreased platelet activation (mean 5·84 *v*. 6·60 %) and plasma CRP concentrations (mean 0·76 *v*. 0·97 mg/l) in the treatment group *v*. placebo group
Karlsen *et al.* ^(^ ^142^ ^)^	120 men and women, age 40–74 years	Anthocyanin extract from bilberries and blackcurrant	300 mg/d for 3 weeks	Decreased plasma concentrations of IL-8 (25 %), IFN-α (15 %) and RANTES (15 %) in the treatment group *v*. placebo group, but no results observed for CRP
Oyama *et al.* ^(^ ^92^ ^)^	Thirty healthy smokers, mean age 37 years	Green tea catechins	Control dose 0 mg, middle dose 80 mg and high dose 580 mg; 2-week intervention	Decreased concentrations of 8-OHdG (20 %), IL-6 (42 %) and soluble Fas (25 %) in the high-dose group *v*. catechin-free group at day 14, but no results observed for IL-1β
Widlansky *et al.* ^(^ ^143^ ^)^	Sixty-six men and women, average age 54 years	Black tea	900 ml/d for 4 weeks	No effects observed for plasma CRP and urinary 8-OHdG concentrations
Mellen *et al.* ^(^ ^144^ ^)^	Fifty men and women with coronary disease, mean age 58 years	Muscadine grape seeds	1300 mg/d for 4 weeks	No effects observed for plasma CRP and IL-6 concentrations
Heinz *et al.* ^(^ ^145^ ^)^	120 women, age 30–79 years	Quercetin	500–1000 mg/d for 12 weeks	No effects observed for plasma IL-6 or TNF-α concentrations

CRP, C-reactive protein; TNF-R2, TNF receptor 2; IFN-α, interferon-α; RANTES, regulated on activation, normal T-cell expressed and secreted; 8-OHdG, 8-hydroxydeoxyguanosine.

Although the effects of flavonoids were originally ascribed to an antioxidant action, it is now clear that levels achieved in biological tissues may not be sufficient to act in this way. Evidence indicates that flavonoids are capable of acting in a number of other ways that may result in their targeting of inflammation, including (1) the modulation of intracellular signalling cascades that control neuronal survival, death and differentiation; (2) an impact on gene expression and (3) interacting with the mitochondria^(^ ^95^ ^–^ ^98^ ^)^. In particular, emerging evidence suggests that dietary flavonoids may exert neuroprotective effects by suppressing the activation of microglia, which mediate inflammatory processes in the CNS (see the earlier section). Although rather complex, the main anti-inflammatory properties of flavonoids include (1) an inhibitory role in the release of cytokines, such as IL-1β and TNF-α, from activated microglia; (2) an inhibitory action against inducible NO synthase induction and subsequent NO production in response to glial activation; (3) an ability to inhibit the activation of NADPH oxidase and subsequent generation of reactive oxygen species in activated glia; and (4) a capacity to down-regulate the activity of pro-inflammatory transcription factors, such as NF-κB, through their influences on a number of glial and neuronal signalling pathways^(^ ^99^ ^,^ ^100^ ^)^. However, almost all mechanistic studies have been carried out *in vitro* at rather supraphysiological concentrations, with limited research on animal models and scarce data from human RCT.

### Early-life nutrition and inflammation

During development, the human embryo and fetus undergo an enormously complex series of changes in both cell type and cell number. Each of these changes takes place in a strictly choreographed series, and disruption of the process can lead to dramatic and long-lasting consequences. There are many recent summaries of the processes involved^(^ ^101^ ^)^. In humans, this was most clearly demonstrated in the Second World War, when Dutch women were placed under famine conditions following a railway workers' strike (the Dutch Hunger Winter)^(^ ^102^ ^,^ ^103^ ^)^. Studies on the offspring of women who were pregnant at this time have shown clearly that women who were pregnant in the first trimester gave birth to babies who would go on to develop a much wider spectrum of health problems than babies born to women who were in the second or third trimester, though these offspring still would continue to show health problems^(^ ^104^ ^)^.

Factors other than undernutrition can also have both short- and long-term consequences. Of particular relevance, obese women give birth to babies with a higher risk of both small for gestational age and large for gestational age, of complications at birth and of developing the MetS^(^ ^105^ ^,^ ^106^ ^)^. All these cannot be explained by postnatal events, and are at least partly explained by the phenomenon known as ‘fetal programming’ or ‘developmental programming’^(^ ^107^ ^,^ ^108^ ^)^. This hypothesis states that nutrition-related exposures *in utero* ‘programme’ the baby to expect a postnatal nutritional environment, and if a different one is experienced, then there is a risk of the development of metabolic complications. There have been refinements to the basic hypothesis and to our understanding of the mechanisms involved^(^ ^109^ ^–^ ^112^ ^)^; however, the fundamental observations remain unchanged and unchallenged. How these associations are mediated is not yet clearly demonstrated, but several hypotheses are being tested. There is substantial support for nutrition altering the epigenetic profile of the offspring, including hypermethylation of cytokine receptors. Evidence indicates that low Fe status at birth, which is associated with impaired lung function in children, can result in reduced nephron number and decreased levels of cell-cycle enzymes^(^ ^113^ ^)^, suggesting that nutritional deficiency during a critical phase of development can inhibit organ growth. This fits with data showing that thymus growth is reduced, and that this leads to changes in the cytokine profile.

Maternal obesity also has dramatic effects on pregnancy outcome. Again, there are many detailed reviews dealing with this topic^(^ ^107^ ^)^. The mechanisms seem to involve inflammatory responses, and increased cytokine levels have been reported in the placenta and cord blood of babies born to obese mothers. Whether, in humans, the placenta alone is responsible is not clear, and it is quite likely that adipose tissue itself, which becomes infiltrated with macrophages, will produce increased amounts of pro-inflammatory cytokines^(^ ^114^ ^)^. The situation becomes more complex in obesity, because in addition to the cytokines, or possibly because of the cytokines, inflammation results in changes in Fe metabolism^(^ ^115^ ^)^, and there is abundant evidence to show that decreased Fe status during pregnancy has adverse effects on the offspring^(^ ^116^ ^–^ ^119^ ^)^. Obesity results in increased hepcidin production^(^ ^120^ ^,^ ^121^ ^)^. Hepcidin is a negative regulator of Fe absorption^(^ ^122^ ^)^, and lower Fe status in the mother before birth is associated with an increased risk of wheezing in the children (W Bright, G Devereux, HJ McArdle, unpublished results). Thus, decreased Fe status may be an additional risk factor in obese mothers.

## Translating research into public health benefit and novel products

### Biomarkers of inflammation in human nutrition studies

As explained previously, inflammation is a normal process, and there are a large number of cells and mediators involved; measurement of these is often used as a ‘biomarker’ of inflammation, i.e. an indicator that inflammation is occurring. These cells and mediators are largely involved in, or are produced as a result of, the inflammatory process, irrespective of the trigger or its location in the body, and are common to all inflammatory situations^(^ ^1^ ^)^. To monitor inflammation in a meaningful way, the markers used must be valid: they must reflect the inflammatory process under study and must be predictive of future health status. The range of potential biomarkers of inflammation was considered by an Expert Group of ILSI Europe, with the aim of identifying robust and predictive markers, or patterns or clusters of markers, which can be used to assess inflammation in human nutrition studies in the general population; markers indicative of a specific inflammatory pathology (e.g. rheumatoid arthritis) and/or in less accessible tissue sites (e.g. in lung lavage fluid or in intestinal biopsy material) were not considered to be relevant to more healthy populations^(^ ^7^ ^)^. Currently, there is no consensus as to which markers best represent low-grade inflammation^(^ ^6^ ^)^, or differentiate between acute and chronic inflammation or between the various phases of inflammatory responses^(^ ^7^ ^)^. Therefore, a range of blood cellular markers (e.g. total leucocytes, granulocytes and activated monocytes) and soluble mediators (cytokines and chemokines (TNF, IL-1, IL-6, IL-8, CC chemokine ligand 2 (CCL2), CCL3, CCL5), adhesion molecules (vascular cell adhesion molecule-1, intercellular adhesion molecule-1, E-selectin), adipokines (adiponectin) and acute-phase proteins (CRP, serum amyloid A, fibrinogen)) are frequently measured. Some of these are associated with future risk of CVD and with cardiometabolic health^(^ ^1^ ^,^ ^6^ ^,^ ^7^ ^)^. However, there are several key issues concerning the use of these markers as determinants of low-grade inflammation. First, they are non-specific acute-phase response and pro-inflammatory response markers, and, by themselves, do not represent metabolic low-grade inflammation. Second, even in healthy individuals, there is wide variation in the measurements made. This is because there are a number of modifying factors that affect the concentration of an inflammatory marker at a given time. These modifying factors include age, diet, body fatness, physical fitness and genetics, among others^(^ ^1^ ^)^.

One can question whether static measurements of single or complex biomarkers are truly informative about health status, reasoning from the concept that health is defined by the ability to adequately adapt to everyday challenges^(^ ^123^ ^)^. Measuring the concentration of inflammatory markers in the bloodstream under basal conditions is probably less informative and relatively insensitive compared with measurements of the concentration change in response to a challenge. A number of inflammatory challenges have been described. These include an oral glucose load^(^ ^80^ ^)^, an oral fat load^(^ ^124^ ^,^ ^125^ ^)^, acute exercise, administration of bacterial LPS^(^ ^126^ ^)^, exposure to UV irradiation^(^ ^127^ ^,^ ^128^ ^)^ and vaccination. Although each of these challenges has been used in nutritional studies, many are poorly standardised, limiting the comparisons that can be made. Most often, the markers measured in response to challenges are those mentioned earlier in the context of static basal measurements. Currently, a number of large European consortia, i.e. PhenFlex (http://www.nugo.org/everyone/42 701/7/0/30), NutriTech (http://www.nugo.org/nutritech) and BioClaims (http://bioclaims.uib.es), are developing and validating the metabolic challenge test concept for application in the assessment of health status, including the study of inflammatory process markers^(^ ^129^ ^)^.

The past decade has seen huge growth in innovation in ‘omics’ technologies that provide enormous opportunities for high-throughput biological sample characterisation, with patterns and clusters of markers (signatures or fingerprints) emerging as robust biomarkers of inflammation^(^ ^89^ ^,^ ^130^ ^)^. The enormous challenge in this era of big data is making biological sense of different levels of data, including the transcriptome, proteome, metabolome and clinical chemistry data. Novel data analysis methodologies, such as machine learning, offer large potential for identifying relevant data for specific biological outcomes based on complex multidimensional datasets^(^ ^131^ ^)^. In addition, bioinformatic tools have been developed to interpret these complex data in the context of existing biological knowledge in the literature and databases, also termed network biology^(^ ^132^ ^,^ ^133^ ^)^. These technologies will be instrumental to the discovery of relevant biomarker signatures that reflect ‘low-grade inflammation’ based on inflammatory response networks connected to organ-specific metabolic derailment.

With the coming of age of the ‘omics’ technologies and bioinformatic tools, a large increase in the number, specificity and sensitivity of candidate biomarkers of inflammation can be expected in the next decade^(^ ^134^ ^)^. A screening of the ‘Thomson Reuters Integrity^SM^ Biomarker Database’ reveals that as of May 2014, 945 candidate biomarkers of inflammation have been described, of which only seventeen, including CRP, TNF-α, serotransferrin and erythrocyte sedimentation rate, have been developed into biomarker assays approved and recommended by regulatory bodies for use in clinical studies. This represents the classical biomarker gap: many candidate biomarkers are identified based on preclinical and clinical studies; however, due to relatively limited efforts in validation and assay development, these are subsequently not further developed^(^ ^135^ ^)^. To accelerate biomarker development, a paradigm shift in this area is needed; instead of single companies developing a single biomarker assay, pre-competitive collaborations between different industrial, academic, and research and technology organisations have the advantage of a more efficient development process time- and cost-wise, by combining a wide diversity of expertise, in the development of a harmonised, standardised and accepted assay. In these consortia, ideally, companies from nutrition, pharma and diagnostics join forces in a pre-competitive way.

A major concerted effort should comprise (1) the discovery of context-based biomarker signatures for the assessment of the status of low-grade inflammation, (2) the development of challenge tests that determine the inflammatory response functionality in the context of metabolic stress-induced low-grade inflammation, and (3) the development of the identified biomarkers towards application in a clinically accepted assay, with normative data.

### Low-grade inflammation and health claims

The European Food Safety Authority (EFSA) guidance document on scientific requirements for health claims related to gut and immune function^(^ ^136^ ^)^ specifically states that chronic inflammation is associated with the development of a number of diseases, and that ‘altering levels of markers of inflammation might indicate a beneficial effect in the context of “a reduction of disease risk claim”, if it can be demonstrated that altering the levels of inflammatory markers is accompanied by a reduced incidence of a disease for a specific dietary intervention’. No additional specificity is added for chronic low-grade inflammation. At present, the European Union health claim register (http://ec.europa.eu/nuhclaims) does not contain any authorised or non-authorised health claims that specifically address the health benefit area of suppression or control of low-grade inflammation.

**Fig. 2:**
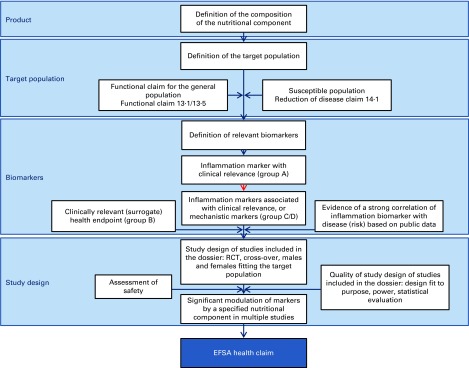
Schematic of topics to be addressed when building a dossier for a European Food Safety Authority (EFSA) health claim on control of chronic low-grade inflammation. The blue boxes indicate the main topics to be addressed; the white boxes state the actual content topics. Building a strong EFSA health claim dossier requires (1) a definition of the composition of the nutritional component including manufacturing procedures in scope and out of scope for the claim, (2) a clear definition of the target population, being the general population or a specific subpopulations at risk, including the defining parameters, (3) a definition of biomarkers measured to assess the health effects of the nutritional component, including a description of the proof of clinical relevance, or the clinical validity of the combination of inflammation biomarkers and related clinically relevant biomarkers for health benefit endpoints associated with the health claim, and (4) a full description of clinical study design for all studies included in the dossier, including statistical power analysis and safety evaluation. The red arrow indicates the primary hurdle for functional health claims in the area of chronic low-grade inflammation, which is the lack of (combinations of) inflammation biomarkers with established and therefore accepted clinical relevance. This is primarily the consequence of inflammatory responses being non-specific normal physiological responses to tissue damage, and discrimination between normal and abnormal levels or combinations has not been well established in relation to chronic low-grade inflammation. The description of the classification of clinical relevance of biomarkers (categories A–D) was adapted from Albers *et al.* ^(^ ^137^ ^)^. RCT, randomised controlled trial. (A colour version of this figure can be found online at http://www.journals.cambridge.org/bjn).

To build strong health claims on nutrition for improving inflammation control in the future, one of the key focus areas should be the need for clinically relevant prognostic marker(s) or marker signatures that reflect the inflammatory state in a context-specific manner, which have been well validated and for which a robust standardised assay is available. The lack of health claims is probably attributable to the fact that, although numerous biologically plausible mechanisms have been established to explain inflammation–disease associations, no single biomarker or cluster of biomarkers of inflammation has yet been robustly demonstrated to be sufficiently predictive of future disease. Based on the EFSA guidance on this topic^(^ ^136^ ^)^ and the classification of candidate biomarkers as described by the expert group of ILSI Europe^(^ ^137^ ^)^, the suggested strategy for building a EFSA health claim dossier (Fig. 2) comprises (1) a definition of the composition of the product; (2) a well-founded selection of the target population; (3) the selection of a clinically relevant composite biomarker panel representing inflammation as well as the selected health benefit (or disease risk) endpoints; and (4) a number of sufficiently powered and well-controlled human studies assessing the effect of the test material (nutrient, food, product) on the relevant biomarkers in the relevant target population.

## Summary and suggestions for the way forward

Inflammation is a normal component of host defence; however, elevated unresolved chronic inflammation is a core perturbation in a range of chronic diseases and is an important determinant of the pathological impact of excess adiposity. Cell, animal and human epidemiological studies have identified a number of potential diet derived anti- and pro-inflammatory components, some of which have been discussed here; this topic has been dealt with more extensively elsewhere^(^ ^1^ ^,^ ^6^ ^,^ ^7^ ^)^. Available human RCT evidence is more limited and sometimes conflicting or inconsistent, in part attributable to under-powered studies where inflammation was not specified as a primary study outcome. Furthermore, research tends to take a reductionist approach and examine the impact of individual dietary components in isolation, despite the identification of numerous potential diet-derived anti-inflammatory and inflammation-resolving bioactive compounds, with likely additive or synergistic effects. There is a need to take a more holistic approach and consider the impact of combinations of components of foods and dietary patterns, with a likely greater overall benefit than each single component might have on its own. Moreover, although it is evident that the inflammatory response is highly variable, a full understanding of the source of heterogeneity is distinctly lacking. More extensive profiling of participants in human studies and consideration of potential key variables such as age, sex, genotype and lifestyle factors in statistical models is needed in order to help understand the aetiology of the variation in both inflammation itself and in its response to dietary change. This approach will also allow for the identification of population subgroups that may particularly benefit from interventions that target inflammation.

Establishing and quantifying reliable, precise diet–inflammation–health associations is reliant on the availability of approved, standardised biomarkers with normative data for use in human observation studies and RCT. Biomarker research is a highly active area with significant advances to be expected in the coming years^(^ ^138^ ^)^. Rather than rely on a limited number of generic markers common to both acute and low-grade chronic inflammation, future inflammation ‘testing’ is likely to involve quantifying clusters or signatures of markers with some tissue specificity. Such biomarkers should generally be measured in the challenged state^(^ ^1^ ^)^, with the choice of the physiological stressor dependent on the tissue, and research question of interest. The biomarkers assessed are likely to include those already typically measured (cytokines, chemokines, soluble adhesion molecules, etc.), but are also likely to include tissue-specific markers and fingerprints based on gene expression profiles (e.g. in blood mononuclear cells), cell or plasma proteomics, and microRNA.

The research focus on the establishment of a robust diet–inflammation–health association is justifiable, considering the substantial role of low-grade inflammation in the pathology of numerous chronic diseases, thereby making it a key future preventative and therapeutic target.
